# Can common dietary assessment methods be better designed to capture the nutritional contribution of neglected, forest, and wild foods to diets?

**DOI:** 10.3389/fnut.2023.1186707

**Published:** 2023-07-06

**Authors:** Jessica E. Raneri, Julia Boedecker, Diego A. Fallas Conejo, Giulia Muir, Giles Hanley-Cook, Carl Lachat

**Affiliations:** ^1^Department of Food Technology, Safety and Health, Faculty of Bioscience Engineering, Ghent University, Ghent, Belgium; ^2^Senior Nutrition Sensitive Agriculture Advisor to the Australian Centre for International Agricultural Research, and Agricultural Development and Food Security Section, Department of Foreign Affairs and Trade, Canberra, NSW, Australia; ^3^Food Environment and Consumer Behaviour Lever, The Alliance of Bioversity International and International Center for Tropical Agriculture (CIAT), Nairobi, Kenya; ^4^Food and Agriculture Organization of the United Nations, Rome, Italy

**Keywords:** biodiversity, diet quality, nutrition, planetary health, food system, diet assessment method, food biodiversity

## Abstract

Food systems are the primary cause of biodiversity loss globally. Biodiversity and specifically, the role that wild, forest and neglected and underutilised species (NUS) foods might play in diet quality is gaining increased attention. The narrow focus on producing affordable staples for dietary energy has contributed to largely homogenous and unhealthy diets. To date, evidence to quantify the nutritional contribution of these biodiverse foods is limited. A scoping review was conducted to document the methods used to quantify the contribution of wild, forest and NUS foods. We found 37 relevant articles from 22 different countries, mainly from Africa (45%), the Americas (19%), and Asia (10%). There were 114 different classifications used for the foods, 73% of these were specifically related to wild or forest foods. Most dietary assessments were completed using a single day qualitative or quantitative 24 h open recall (*n* = 23), or a food frequency questionnaire (*n* = 12). There were 18 different diet related indicators used, mainly nutrient adequacy (*n* = 9) and dietary diversity scores (*n* = 9). Often, no specific nutritionally validated diet metric was used. There were 16 studies that presented results (semi) quantitatively to measure the contribution of wild, forest or NUS foods to dietary intakes. Of these, 38% were aggregated together with broader classifications of ‘traditional’ or ‘local’ foods, without definitions provided meaning it was not possible to determine if or to what extend wild, forest of NUS foods were included (or not). In almost all studies there was insufficient detail on the magnitude of the associations between wild, forest or NUS foods and dietary energy or nutrient intakes or the (qualitative) diet recall methodologies that were used inhibited the quantification of the contribution of these foods to diets. A set of six recommendations are put forward to strengthen the evidence on the contribution of wild, NUS, and forest foods to human diets.

## Introduction

Humanity is off course in meeting global nutrition and non-communicable disease targets by 2030. Despite global efforts, 811 million people are undernourished, one in three people food insecure, 22% of children under 5 stunted, 1.3 billion people suffering from micronutrient deficiencies, and over 40% of adults now overweight or obese (1). Additionally, none of the Aichi Biodiversity Targets have been fully met (2), with biodiversity for food and agriculture declining (3).

The total number of estimated edible plants range from a conservative 7,039 edible species (4) in a broad taxonomic sense to <30,000 (5) yet less than 200 species make a significant contribution to commercial food production, and just nine species account for two thirds of total global crop production (3, 6). The result is an increasingly homogenous global food system reliant on only four crops (rice, potatoes, wheat, and maize) to provide over half of the worlds dietary energy (3, 7). The narrow focus on producing affordable staples for dietary energy, rather than on a diversity of foods to supply micronutrients has contributed to largely non-diverse and unhealthy diets. The effects extend beyond malnutrition and contribute to degrading ecosystems across the world.

Currently, our food system is depleting natural resources, contributing to greenhouse gas emissions and is the primary cause of biodiversity loss globally (8, 9). Despite increasing recognition of the crucial role of biodiversity in maintaining human and planetary health, biodiversity is declining faster than at any time in human history (9). Biodiversity is currently eroding at extremely high rates, with one million plants and animals now threatened with extinction and 10 million hectares of forests are lost every year (10) putting our global ecosystem and resilience at risk. Yet, paradoxically, biodiversity is the lifeline of ecosystems, human and planetary health, and one that supports both environmental and household resilience – economic, food security, and nutrition.

In the context of diets and food, biodiversity is often referring to wild (uncultivated foods gathered or hunted from the natural environment), neglected and underutilised species and sub-species (NUS), and forest foods. Wild foods are food products obtained from non-domesticated species (3) and many wild foods are simultaneously neglected and underutilised species and forests foods. These biodiverse foods are still important sources of food for many rural populations (11), and of particular importance for sustaining Indigenous people’s dietary needs (12). The FAO (3) reports that wild foods contribute to food security via direct consumption – regularly or as an emergency measure in times of scarcity, by being sold to provide income that is reinvested in food purchases or other household needs, and for cultural and recreational use. Harvesting wild food is well-established as an important coping strategy to deal with food insecurity in rural households (13–16). However, wild foods fulfil more than just safety-net functions, and their consumption is often shaped by other factors such as seasonal availability, trade-offs in time, culture and recreation (17).

Food composition data show the often superior nutrient content of many neglected and underutilised species (18). Studies have demonstrated that wild and NUS foods can make significant contributions of micronutrients (e.g., iron), protein and fibre and thus help to attain nutritional security in resource poor settings (17, 19–21). Moreover, forest and tree sourced foods are widely consumed globally and can make substantial contributions to meeting daily fruit, vegetable, meat, fish and micronutrient intake recommendations (22, 23). In addition, NUS are also known to contain substantial amounts of bioactive compounds such as flavonoids that contribute to overall health (19). However nutrient composition can vary greatly across sub-species and varieties, and across geographies due to biophysical differences, which can make the nutritional contribution of these foods even more difficult given very few data are available on biodiverse food nutrient composition.

Compared to major staple food crops, NUS, and wild foods can be more resistant to biotic and abiotic stresses providing more reliable yields under poor soils and adverse climatic conditions (19, 20), thus are considered climate resilient. They often grow spontaneously in marginal and harsh environments that are not suitable for cultivation of main staple food crops, and with minimal or no farm inputs.

Improved food and nutrition security relies on the sustainable use of natural resources and agriculture within planetary boundaries (IPC 2022). Diverse diets that are high in fruit, vegetables, nuts, seeds, and fibre are needed for healthy diets that not only nourish bodies but protect from non-communicable diseases (24). This diet diversity is relevant to multiple dimensions of ‘diversity’ – diverse food groups (fruit, vegetables, meats) but also biologically regarding variation in species and varieties of food types both across and within food groups (rice, quinoa, buckwheat), i.e., food biodiversity (25).

Biodiversity and specifically, the role that plants, including wild, forest and NUS foods might play in diet quality is gaining increased attention (26). To date, however, the evidence to quantify the nutritional contribution of forest, wild, and NUS is patchy and overall limited. Large-scale dietary intake surveys often focus only on the most commonly consumed foods (e.g., food frequency questionnaires – FFQ) and rarely assess locally available food, let alone wild, forest and NUS foods as few individual or population-representative methods are designed to capture taxonomical detail on foods consumed (27–29). Furthermore, inconsistent recall periods and frequencies of data collection contribute to the difficulties in capturing wild, forest and NUS foods that are often highly seasonal and availability can change across geographies, even within a country or region. The complexity of capturing day-today variability has been shown even at the higher aggregated food group level (30), which has easier and more widely adopted methods for data collection.

There is a breadth of studies that document the depth of food biodiversity available in local food systems (31–33). During qualitative assessments communities report that these are resources utilised, yet often these do not appear in dietary intake assessments or national food-based dietary guidelines. Diverse methods for dietary intake assessments exist, and more recently, tools to motivate the scientific community to generate, collect, compile and disseminate more data on biodiversity in foods (34) are available. However, existing dietary intake assessment tools (including lack of food composition tables), methods and skills are inadequate, time-and labour-intensive, expensive and unlikely to capture the diversity fully and systematically in diets.

The absence of global evidence and data on the contribution of wild, forest and NUS foods to diets worldwide, challenges the development of evidence-based policy recommendations that advocate for more biodiverse food systems for better nutrition and more sustainable food production. The present perspective draws on a scoping review of available literature aiming to (i) describe the dietary assessment methods used to capture the nutritional contribution of forest, tree, wild, and NUS, (ii) quantify the contribution of wild, forest and NUS foods to diets, and (iii) provide recommendations for researchers, development program managers and policy makers for the use of more accurate dietary assessments that adequately capture and demonstrate the importance of biodiverse foods to diet quality and nutrition.

## Methods

A systematic process was followed for scoping reviews and papers were extracted from MEDLINE (through PubMed). Although a search of various databases is recommended to provide a comprehensive systematic review of published literature, we restricted our search to MEDLINE whilst utilising a broad syntax to provide a cross-section of how researchers have assessed nutritional contribution of neglected, forest and wild foods in dietary assessment, as basis for methodological considerations and recommendations. The objective of the literature review was to provide an obtain a cross-section of peer reviewed literature on the topic to inform a further reflection on dietary assessment methods. Although MEDLINE currently indexes most of the journals that publish research on nutrition and dietary intake, the present review is hence not an exhaustive review of the literature on the topic.

As there is no agreed botanical definition of what a “forest,” “tree,” “wild,” or “neglected” food is, a comprehensive free text search was used. The free text search extracted all relevant papers with any of these words in the publicly available fields (e.g., title, abstract, authors, affiliations, and keywords). There were no restrictions regarding the date of publication in the search syntax. Table 1 describes the components of the search syntax and SI1 details the exact syntax and number of unique titles retrieved. Two searches were conducted, one to generate results on the contribution from forests or trees, and the other specifically on wild or neglected foods. The results of these were initially reviewed individually and were then combined after mapping. The database was searched on 8/3/2021 and data were extracted on this date. The scoping review was conducted using the relevant PRISMA extension (35).

**Table 1:** Search syntax of the scoping review.

Parameter	Description
Population	Restricted to studies on humans. Only healthy, free-living populations will be used. Studies in clinical settings or allergens will be excluded. There are no geographical limitations.
Interventions	Inclusion criteria: any study with objective of measuring participants dietary intake from forest, tree, wild, or neglected foods over a reference period.
Comparators	All comparators are used.
Outcomes	Inclusion criteria: any measure of dietary intake.
Study design	Inclusion criteria: all study designs, published in English. Exclusion criteria: editorials, letters, comments, conference reports, and expert opinions will be excluded.
Year	There were no restrictions regarding the date of publication.

All retrieved titles were imported in Mendeley and screened for eligibility based on their titles (DFC). When in doubt, the titles were kept for further screening. Abstracts of retained titles were assessed by two researchers (DF and JB). Finally, full-text papers were examined, and relevant information was extracted and mapped using a pre-established data extraction template (DFC & JB) (SI2). Full text papers were excluded from the review if they reported on nutrition or health outcomes not directly related to diet quality or micronutrient status measurements (e.g., cancer or anthropometrics). After data were mapped and extracted, another researcher reviewed these for the final inclusion assessment (JR). To understand how papers characterised “forest,” “tree,” “wild,” or “neglected” foods, a search was conducted on how these foods were characterised and referred to, specifically in methodology and results sections. A word cloud online tool was used to compute and visualise the frequency of the terms used to classify foods [Free Word Cloud (36)].

## Results

**Figure 1:**
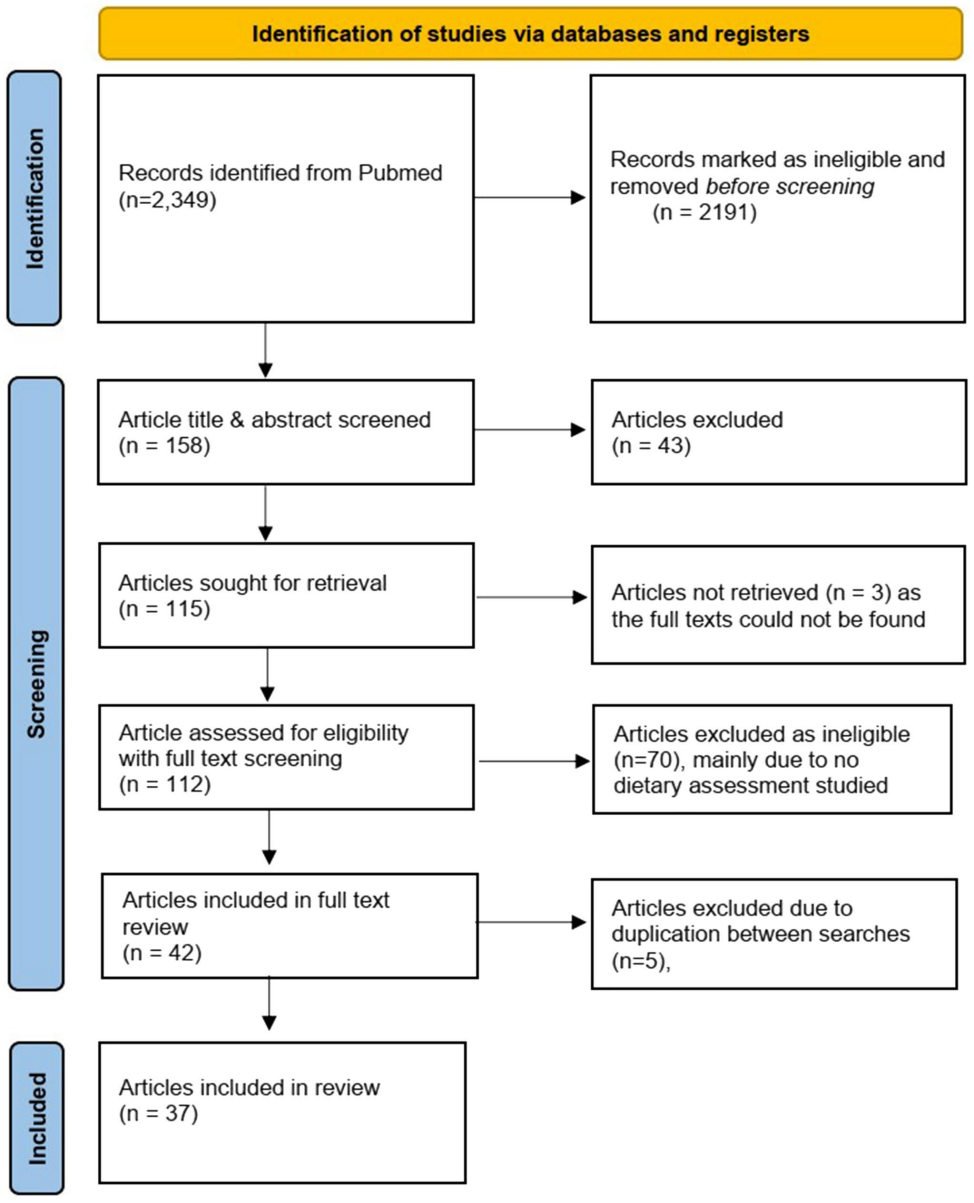
Flow chart.

In total, 2,349 papers were retrieved (SI3). After title and abstract screening, 115 full text papers were identified as meeting the search criteria. After screening the full texts, 42 papers were included for data mapping. Five papers were excluded at this stage as they were duplicated in both syntax search results, finally resulting in 37 papers that were included in the scoping study (Figure 1).

There was a clear geographic bias, from the 37 studies included, there were 22 different country populations represented. Of this, 45% of papers were from African countries compared to 19% from the Americas, 10% from Asia, 7% from Europe and only one paper from the Pacific. Of these, all but eight papers were from rural areas of low-and middle income-countries (Table 2).

**Table 2:** Summary of studies examining the nutritional contribution of food biodiversity.

Reference	Country	Study period	Sample size (*n*)	Study design	Dietary assessment method
Ahenkan and Boon (37)	Ghana	April–July 2009	Household heads (200)	Cross-sectional	Structured questionnaire: no specific dietary assessment method specified
Belahsen et al. (38)	Morocco	Not specified	Women and men from both rural (35%) and urban (65%) areas (not specified)	Cross-sectional	Quantitative FFQ, including 130 foods from 9 food groups; focus group discussions to collect recipes and their preparation
Belanger et al. (39)	India	September–December 2017	Women (100)	Cross-sectional	Qualitative 7-day FFQ; quantitative 24-HR
Benhura and Chitsaku (40)	Zimbabwe	January, May, and August 1988	Households (438)	Cross-sectional	7 consecutive qualitative, mainly open ended 24-HRs
Blanchet et al. (41)	Canada	February–August 2018	Women (70%) and men aged >19 years (265)	Cross-sectional	Quantitative 24-HR; second 24-HR among 19% of participants
Campbell (42)	Zimbabwe	August 1983 (dry season)	Households (225)	Cross-sectional	Qualitative 24 h recall; non-standardised method to recall foods consumed more frequently than weekly, but not captured in 24-HR period
do Nascimento et al. (43)	Brazil	January 2008 and January 2010 (end of the dry and rainy season)	Women (66%) and men (68)	Cross-sectional	Two ‘modified’ qualitative 24 h recall in which participants cited the plants consumed in the last week
Dop et al. (44)	Tunisia	November 2014–October 2015	Women aged 20–49 years (584)	Cohort study	4 qualitative 3 month FFQs
Dounias et al. (45)	Indonesia	June, September, and December 2003; March and July 2004 and September–November 2004	Households (43) and Households (20)	Cross-sectional	2–4 consecutive comparative quantitative 24 h food consumption surveys; 20 day semi-quantitative food survey
Fungo et al. (46)	Cameroon	May 2012	Non-pregnant and non-lactating women aged >18 years (279)	Cross-sectional	2 non-consecutive quantitative 24-HR; qualitative 7 day recall
Golden et al. (47)	Madagascar	March 2008–February 2009	Households (28) with children aged 5–12 years (77)	Prospective cohort	Self-administered daily quantitative diet calendar, recording the type (i.e., chicken, duck, fish, beef, pork, or species of wildlife) consumed by the household; observations permitted the calculation of a mean proportion of stew typically consumed by individuals by summing all spoonfuls consumed and then calculating an individual’s allotment
Golden et al. (48)	Madagascar	July 2013–March 2014	Households (152) with individuals of both sexes aged 6 months–73 years (719)	Prospective cohort	Quantitative daily household diet records (i.e., female head of household recorded the weight of all food that was cooked in the household for three meals per day every day); out-of-household consumption data were reported monthly, with frequency of consumption during the month recalled by the respondent.
Kaufer et al. (49)	Micronesia	Baseline: June–August 2005; Endline: June–August 2007	Women (40)	Single-group, pre-post test	Quantitative 7 day FFQ, including 33 food items and 200 sub-items; 2 non-consecutive quantitative 24-HR
Kent and Dunn (50)	Botswana	May–August 1988; May–August 1989	Household members (#not specified)	Cohort study	Daily qualitative 24-HRs
Kolahdooz et al. (51)	Canada	Not specified	Non-pregnant and non-lactating women (80%) and men aged ≥19 years (213)	Cross-sectional	Population-specific, culturally appropriate quantitative 1 month FFQ
Kruger et al. (52)	South Africa	Not specified	Women aged 18–57 years (13)	Cross-sectional	Adapted 1 week food coping strategy questionnaire throughout 1 year during 5 seasonal periods: early summer, late summer, autumn, winter, and spring
Liberda et al. (53)	Canada	2005–2009	Women (57%) and men aged >8 years (1,429)	Cohort	Quantitative 24-HR and traditional FFQ, including 67 food items
Manios et al. (54)	Greece	February–March 2003 (wild green season); August–September 2003	Women (72)	Cross-sectional	3 non-consecutive quantitative 24-HRs
Mansuri et al. (55)	Canada	2003–2005	Women (59%) and men aged >12 years (445)	Cross-sectional	Quantitative 3 month FFQ, including 36 food items; adapted structured 1 month vitamin D questionnaire on sun exposure and dietary sources of vitamin D-containing foods
M’Kaibi et al. (56)	Kenya	Not specified (dry and rainy seasons)	Children aged 24–59 months (525)	Cross-sectional	2 non-consecutive quantitative 24-HRs per season
Ndaba and O’Keefe (57)	South Africa	October–November 1983	Women (66%) and men (869)	Cross-sectional	Qualitative 1 month FFQ
Ntwenya et al. (58)	Tanzania	February–May (rainy season); September–October 2011 (post-harvest season)	Households (307)	Cross-sectional	Qualitative household 24-HR
Oduor et al. (59)	Kenya	September–October 2014; March–April 2015	Children aged 12–23 month (634)	Cross-sectional	2 non-consecutive quantitative 24-HRs
Ogle et al. (60)	Vietnam	1997–1998 (rainy seasons)	Women aged 19–60 years (196)	Cross-sectional	Adapted quantitative 7 day FFQ
Penafiel et al. (28)	Ecuador	March–May 2011	Non-pregnant and non-lactating women (178)	Cross-sectional	2 non-consecutive quantitative 24-HRs
Powel et al. (61)	Tanzania	March–May (rainy season); September–October 2009 (dry season)	Children ages 2–5 years and their mothers (274 rainy season; 129 dry season)	Cross-sectional	Qualitative 7 day FFQ; 2 non-consecutive quantitative 24-HRs (rainy season) Qualitative 7 day FFQ; Quantitative 24-HR (dry season)
Powell et al. (62)	Morocco	March–April 2005	Households (103)	Cross-sectional	Qualitative 7 day household FFQ
Rao et al. (63)	India	Not specified	Children aged 1–6 years (1,401)	Cross-sectional	Quantitative 24-HR
Robinson and Remins (64)	Central African Republic	June–August 2012	Women >18 years (60)	Cross-sectional	Qualitative 24-HR; qualitative 7 day FFQ
(65)	Ecuador	March 2009	Children aged 2–6 years (160)	Quasi-experimental	Semi-quantitative FFQ
Skreden et al. (66)	Norway	1999–2008	Pregnant women (55,056)	Cohort	Quantitative FFQ, including 225 food items
Tata et al. (67)	Cameroon	July 2013, February 2014, and February 2016	Women (247)	Cross-sectional	3 qualitative 24-HRs
Taylor et al. (68)	United Kingdom	2008–2010	Adults <65 years (#1031); children >1.5 years (#1095)	Cross-sectional cohort study	4d quantitative diet diary
Termote et al. (69)	Democratic Republic of Congo	July–September 2009	Non-pregnant and non-lactating women (492)	Cross-sectional	2 non-consecutive quantitative 24-HRs
Termote et al. (70)	Kenya	February–June (dry season); July–August (wet season) 2012	Women, pregnant women, lactating women, and children aged 6–23 months (not specified)	Cross-sectional	Ethnobiological inventory by focus group discussions; individual interviews and focus group discussions to identify culturally acceptable average consumption frequencies of all foods
Van Dijk et al. (71)	Cameroon	September 1997–October 1998	Households (30)	Cross-sectional	Daily records on the product flow of non-timber forest products and agricultural produce for a period of more than 1 year
Wesche and Chan (72)	Canada	1997–2000	Women and men aged >15 years (not specified)	Cross-sectional	Quantitative 3 month FFQ

24-HR, 24 h dietary recall; FFQ, food frequency questionnaire.

All studies collected primary data. In total, there were six different study designs used. Most studies (*n* = 27) were cross-sectional, with few longitudinal or in-depth case studies (four and two respectively). There were five intervention studies and only one provided sufficient information on observed effect coefficients. Women of reproductive age and children were most commonly the target population (57%). All other studies focused on household consumption generally and even when data were collected for men and women, the utilisation of foods was presented at an aggregated household level. No studies captured the consumption of all household members to allow for gender-disaggregated or intra-household analysis.

Sample sizes varied greatly from 13–55,000, and most studies had sample sizes between 200–600 respondents. In three cases no sample size was specified. Studies rarely presented justifications on sample size numbers or the relevance of the study power to be able to measure effects or relationships on diet quality.

**Figure 2:**
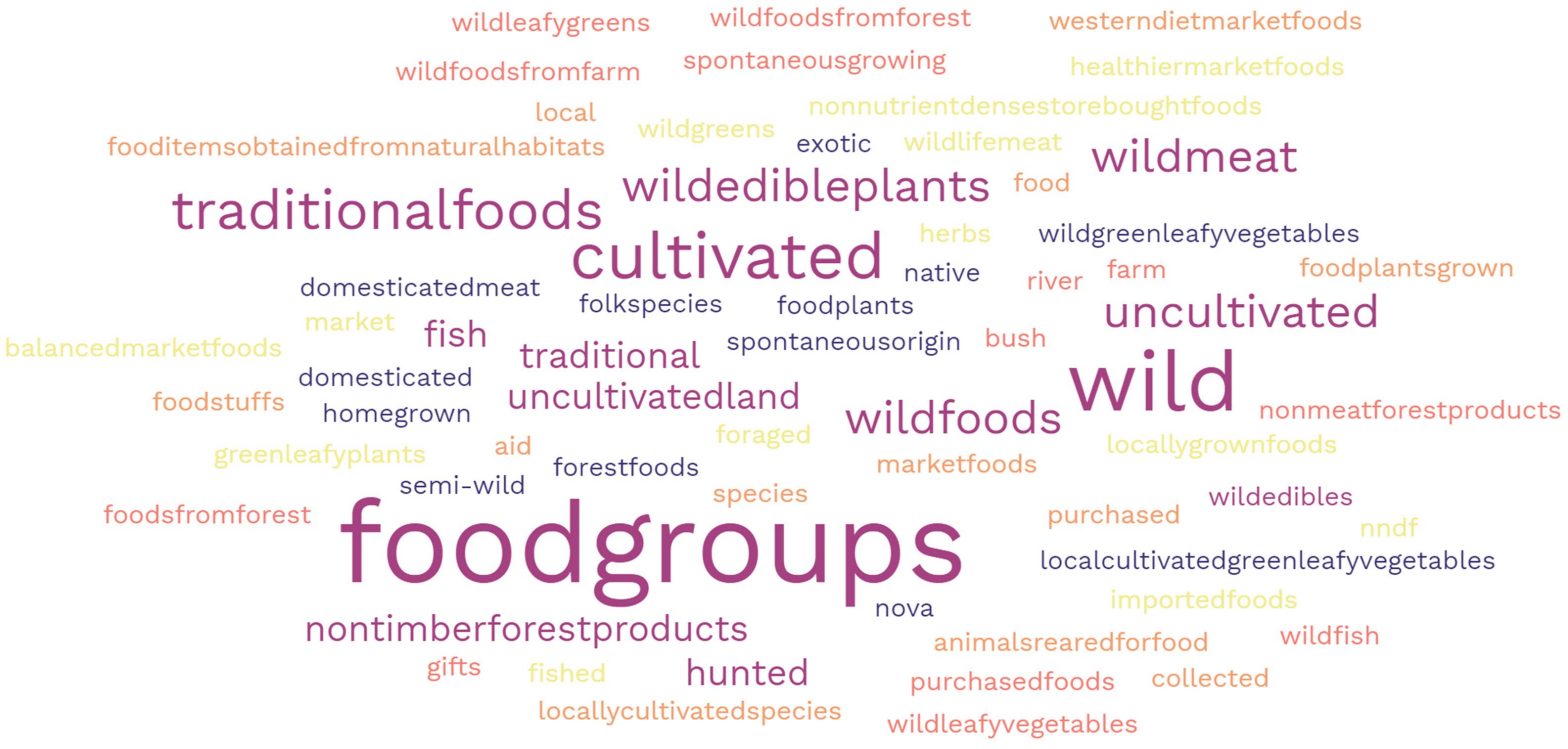
Word cloud representing the frequency of different food classification terms used to categorise wild, NUS, and forest foods.

There were 114 different terms used to classify the foods reported in the methodology and results sections of papers, 73% of these were specifically related to wild, NUS or forest foods in some way. Foods were most frequently classified based on their nutritional function food group (greens, vegetables, fruits etc.) (9%), followed by wild (6%), and traditional (5%) (Figure 2). Foods that were identified in some way related to as wild and forest (e.g., semi-wild, forest, store-bought, and cultivated) were mainly not accompanied by a definition – with 31 classifications undefined. In one case, traditional food also included medicines or teas, as well as generally meals/recipes instead of individual foods, and other where it included both cultivated and uncultivated foods. Despite including the term ‘neglected’ in the search, no papers in the scoping study included the foods that were defined or classified as such.

Most (*n* = 23) dietary assessments were completed using a qualitative or quantitative open 24 h recall (24HR) for a single day only, the next most used methodology was a food frequency questionnaire (FFQ) (*n* = 12) (Table 2). Most commonly, a FFQ was used for a 7 day period (*n* = 10), and FFQs for other recall periods varied from 3 days to 1 year. FFQs referred to a large variation in the number of pre-determined foods included in lists from 36 up to 190 foods. In most cases, the number of foods was not disclosed. It was very common that authors referred to their diet recall methods as ‘adapted’, however details were often not provided on how and in which way they were adapted (43%). Most studies utilised only one dietary intake assessment method (71%), and mainly used a single recall, with only five studies conducting repeated dietary recalls on non-consecutive days and two across different seasons.

Non-standardised, non-validated, or non-articulated (meaning, insufficient detail provided about the recall) methods were used in 11 studies. Information regarding seasons during data collection were inconsistently reported, with only 30% of papers clearly articulating a seasonal period as rainy, dry, post-harvest, summer, winter, etc. Others simply mentioned the months or periods when the recall, or even more commonly the study overall, was conducted. In most cases, the relevance or impact of the season was not put within context of the results.

There were 18 different diet related indicators used (SI4) and all studies used more than one metric. The most used indicators were nutrient adequacy and dietary diversity scores (nine studies each), followed by food variety score and frequency of consumption (seven studies each) and mean nutrient intake (six studies). Food security and anaemia were two outcome measures that were slightly outside the scope of the study but were nonetheless included. There was a combination of both validated and unvalidated/standardised metrics used. Often, no specific nutritionally validated dietary quality metric was used and instead frequency, prevalence, or average consumption of a food or food group was reported (43%).

In over half the studies (57%), diet related results did not specifically report any reference to wild, forest or neglected foods – but rather an overall description diet quality or aggregated frequency of consumption of foods (e.g., traditional). In some cases, consumption was assumed from production yields or frequencies, or simply reporting of a food’s ‘use’ (*n* = 2). Specifically, regarding wild, forest and neglected foods in the diets, the most used metric was frequency of their use and prevalence of their consumption (generally).

In most cases, diet recall results presented only reflected frequency or presence of the food in the diet without contextualising what this meant in terms of contribution to diet quality and nutrition. There were 16 studies that presented results (semi) quantitatively to measure the contribution of wild, forest or neglected foods to diet outcomes (SI5). Of these, 38% were aggregated together with broader classifications of ‘traditional’, or ‘local’ foods which were either defined as including both cultivated and wild or were simply undefined which meant it was not possible to quantify the dietary contribution of wild, forest and NUS foods either individually or collectively in a meaningful way (SI6).

## Discussion

The studies identified in this scoping exercise reported that wild, forest and neglected foods are consumed in certain contexts and quantities, yet most failed to present dietary outcome measures reporting based on these food classifications. In almost all studies there was also insufficient information on observed effect coefficients, or the diet recall methodologies used that inhibited the quantification of the contribution of these foods to diet quality.

There were just seven studies (39, 46–48, 61, 64, 69) that were regarded as utilising validated and gold-standard methodologies for measuring and reporting quantifiable contributions of wild, forest or NUS foods to diet quality. These studies utilised more than one dietary intake method (usually a combination of a quantified 24 h recall and a food frequency questionnaire) and repeat recalls on non-consecutive days. These methods require specific nutrition related expertise and can be both financially and time prohibited for many researchers.

As such, it was not surprising that generally, studies did not quantify the contribution of forest, wild or neglected foods to diets or use indicators validated or calibrated to measure different aspects of diet quality. Often, general measures of diet quality were presented (such as diet diversity) without specifically contextualising wild or forest food contribution to these measures. There is a need for novel measures of diet quality and diversity that better capture food biodiversity, and allow for better visibility of wild, forest and NUS foods (73). These would need to be detailed enough to collect detailed descriptive information about the foods consumed that allow for taxonomical identification of foods. Currently, doing so requires significant financial resources to allow for the training of their botanists/agronomists with the botanical knowledge to collect dietary recall data of sufficient quality, or for the training of nutritionist with sufficient skills to understand and document details of foods needed to make taxonomical identifications. There are various novel methodological and mixed-method innovations being tested that attempt to offset some of these costs such as digital photos, apps, citizen science that utilise mobile information communication technology that are being trialled to reduce enumerator burden for dietary recall studies (74). However, these are often not calibrated to capture biodiversity level information and/or are not yet widely validated.

Most often, data were qualitatively described without presenting quantitative data (e.g., frequency of consumption). When quantitative data were presented, there was a high variation in relative importance, and sample sizes were often too small, or it was difficult to evaluate their quantitative significance at a broader population level. In addition, the use of either highly aggregated and undefined food classifications (e.g., traditional) or only specific subcategories (e.g., wild leafy greens or a specific wild animal) were reported. When these terms were used, they were most often undefined. It is imperative that these foods are defined to ensure interpretation of results relative to food and landscape biodiversity. For example, (37) were the only authors that clearly defined forest foods ‘*products used as food and food additives (edible nuts, mushrooms, grass-cutters, snails, fruits, herbs, spices and condiments, aromatic plants, game)*‘, and wild foods were defined by just two authors as those that were uncultivated and/or spontaneous growing (39, 61).

In addition, there was notably a lack of global representation in the studies. Just under half of studies specific to the sub-Saharan African, rural context and only one study in the Pacific. Given that people’s food environments are extremely context specific, and even more so for populations that rely on the natural environment to source wild, forest and NUS foods, there is a clear need for more studies across a wider diversity of geographical and socioeconomic contexts to be able to estimate the quantified contribution on average across a global context. Increasing global representation in the evidence will require a multi-pronged approach, one that includes sensitisation of young nutrition and food system researchers, as well as to those responsible for large scale and national nutrition surveys to the importance of collecting sufficient food biodiversity in population-based surveys. More courses on food biodiversity are needed in nutrition qualifications, as is the integration of nutrition and dietary assessment method courses in rural develop, agronomic and sustainability focused qualifications. Donors need to be responsive to the geographical blackholes within the evidence gaps, and responsive to researchers calling for more flexible funding options and criteria that also, similarly, have geographic criteria that often skews the evidence base available. These limitations made it difficult to measure or ascertain what the specific contribution of biodiverse foods had on diets collectively.

Dietary intake is inherently difficult to measure, and any single method cannot assess dietary patterns or exposure perfectly. In our assessment, we provide evidence that insufficient level of detail is provided to describe the methods used to collect dietary intake information that would enable the reader to replicate the study with the same method or evaluate how effectively they were designed to capture biodiverse foods. The lack of detail provided by studies on the recall methodologies used is concerning given the well documented challenges related to dietary assessment of forest, wild and NUS (25, 34). These challenges include intra-person variability of intakes (which relates to the issue of optimal versus feasible number of days for dietary data collection), facilitated approaches and tools to facilitate the capture of the diversity of (episodic) wild and NUS consumption, nutrient composition, yield and retention factors and seasonal availability.

Methods used, when detailed, were mainly a single 24HR recall (either qualitative or quantitative 24HR recall method and/or FFQ), focused only on the most commonly consumed foods (e.g., FFQ) and had inconsistent recall periods and frequencies of collection. Existing recall assessments are designed as such to be efficient and capture the ‘main foods’ that contribute most to dietary energy intake at a national, regional or even global level. However, these types of tools are not necessarily appropriate for rural populations that rely on highly seasonal wild, forest and NUS species in their diets, especially as a common reporting bias for 24 h recalls is underreporting in general (75). When applied in these contexts without sufficient methodological adaption there are difficulties in capturing wild, forest and NUS foods that are often highly seasonal. Thus, capturing their contribution to the diet and nutrition is reliant on the ‘when’ dietary assessments are conducted, and the skill of enumerators to facilitate articulation of their consumption (i.e., probing).

Very few studies repeated the dietary recall on a non-consecutive day or in another season of the same year which is important to capture seasonal variability of diets. Previous research has indicated that episodically collected and consumed forest foods are unlikely to be recalled (due to memory lapses), whereas bush meat hunting, where legal, is likely to be more memorable than opportunistic collection of fruit and vegetables (39). To minimise measurement error several approaches can be used including using specific visual probes for wild foods, including photobooks that help identify taxonomically different local food biodiversity, including at the sub-species level (e.g., varieties or cultivars) (62, 66, 70). Such approaches may be particularly useful during the lean seasons when these foods might contribute substantially to nutrient intakes.

These 24HR methods also have inherent limitations when used to study the contribution of wild and NUS to diets across seasons (40). To illustrate, methods are mainly focused on short-term intake, but long-term dietary exposure is especially of interest when investigating non-habitual or seasonal consumption. Single 24HR administered in a sufficiently large sample can adequately provide data to estimate population mean intakes, but fails to correctly depict the fraction of the population with usual intakes at the tails of the distribution (70). Seasonal, weekly scheduling and cultural effects on dietary intakes can be accounted for by administering the survey over a longer period of time and including randomly selected days, preferably representative of all seasons of the year (71). Repeated measurement not only requires a lot of resources (e.g., time, funds, enumerators) but survey repetition can also influence a respondents’ diet, due to responder fatigue or learned social approval bias.

Standardised quantitative 24HR methodologies use an open recall rather than a fixed listed method that is used in FFQs (76). Open recalls offer more opportunity for probing and are flexible to accommodate high level of respondent specificity. This can collect information such as a local name of the sub-species information that can identify important micronutrient profile differences (e.g., white vs. orange sweet potato), as well as description of the part of the plant or animal (e.g., sweet potato leaves vs. tuber) consumed. Additionally, the instruments can be easily adapted to include specific probing questions to identify the source of foods to allow for disaggregation on food flows, as well as identify how the landscape is utilised within a local food system and how this influences the food environment.

Most importantly, the 24HR provides an opportunity to generate evidence between locally available biodiversity and the role of biodiverse wild, NUS and forest foods in the diet which are often otherwise missed, if sufficient detail is collected to identify the specific species or variety of food. The following methods and tools can increase reliability of the food biodiversity identified in the dietary intake assessment: using food names in indigenous and local languages; recall tools that are flexible to capture foods with names not on FFQ list (for example) and/or data capture tools and methods that can capture photos (e.g., tablet with camera) of consumed foods for taxonomy identification by a botanist.

Compared to 24HR, FFQs have the added benefit of capturing the relative importance of foods in a diet and have the potential for capturing under-consumed or infrequently consumed foods. However, to do so, the food lists need to be prepared to include these foods. Given that we found most studies that included a FFQ did not specify the number of foods included on the list, and some had as few as 36 foods, it is unlikely that the studies were adequate to truly capture all forest, wild and NUS foods available in the landscape. A short FFQ may underestimate the true inter-person variation in dietary intake, but a very long and detailed one can be time and resource consuming and the burden on the responder may jeopardise data quality. Whilst neither the FFQ or 24HR provide nil methodological trade-offs, the combined application of both 24HR and FFQ can increase the precision of usual dietary intakes (77).

Only a few papers analysed in this study explicitly captured forest or wild food during dietary recalls. However, results were often not presented relating to their consumption relative to diet quality or nutritional contribution to diets. For example, a specific wild or forest food would be reported as a percentage of wild foods consumed, without reference to the contribution of wild foods compared to all foods consumed. This made it difficult to ascertain the nutritional or even relative contribution of these foods to the diet.

As mentioned above, the inconsistent use of food classification terms is problematic. Conflict remains given the lack of standard definitions for the main search terms of interest (wild, forest, neglected, non-wood forest products). In addition, these foods can overlap given their context. For example, a forest food could be cultivated (agroforestry) or managed (e.g., “forest farming”‘), a wild food could also be a forest food (or not) and similarly, a neglected food is very subjective and relative to the context including cultivated foods (78). Despite including the term neglected in the search, no papers in the scoping study included the foods that were defined or classified as such. Likely, this is due to it being combined with ‘wild’ using the ‘OR’ function in the syntax. In addition, ‘neglected’ as a term used within the context of NUS specifically refers to neglected by (mainly) agriculture research, but often is widely used by communities. As such, it also makes sense as to why the use of this term did not result in papers relevant to our nutritionally focused study. It is recommended that future scoping studies use more targeted and specific search terms that capture the types of neglected foods relevant to nutrition, including using the full term ‘neglected and underutilised species’, as well as ‘traditional’, ‘indigenous’ and ‘local’.

Often, dietary recall methods used collected data that could potentially be analysed and reported in a manner to report specifically on the nutritional contribution of forest, wild or neglected foods, however authors did not do so. This could be due to authors not prioritising or understanding the value of presenting dietary intake data as such. Reporting by large, aggregated food groups does not give biodiverse food products the visibility of their relative importance in the diet. There is a need for easy-to-use, validated diet recall methods and diet quality indicators that allow for the disaggregation of food sources to facilitate better understanding of food flows and how local food systems function.

Despite the increasing global attention to biodiverse foods, large-scale dietary intake surveys rarely assess the source of foods, let alone wild, forest and NUS foods. The few methods designed to capture taxonomical detail on foods consumed are rarely used as they are often time consuming, costly and require a certain expertise to implement (34). The large variation in published estimates on the availability of wild foods from 7,039 (79), 10,000 (80) to 30,000 (6) only highlights the lack of clarity around the role of wild, neglected and forest foods in food systems. There is a common trend in studies evaluating biodiversity and nutrition calling for better, validated and easy-to-use dietary intake assessments that capture this information (25, 81) and this study only further adds to the evidence as to the reasons why innovative and novel assessment methods are needed. Widley used methods must evolve beyond capturing the ‘most consumed foods’ – given that ‘most’ is subjective across context – and they need to also document the source of foods consumed. This would easily allow researchers and nutrition practitioners to be able to collect the granularity in dietary assessment data that can allow for better documentation, and assessment of biodiverse foods.

It was unclear to what extent these studies included nutritionist, however given the lack of robust dietary intake assessment reporting it is likely that formally trained nutritionists were not included. Facilitating more interdisciplinary teams in carrying out research that crosses the agriculture-forestry-nutrition nexus could be a helpful solution in tackling some of the problems highlighted in this paper.

The question remains, how can we as nutrition researchers effectively guide policy makers on which foods to increase in production, conservation and food system interventions if we simply do not know to what extent all foods are contributing to diets? Development of effective land-use policies and nutrition interventions must be based on more complete food systems accounting (82). We now have sufficient evidence to know there is an urgent need to support biodiversity in our ecosystems for resilience and sustainability – the same is true for food biodiversity and healthy diets. It’s time for nutrition and dietary intake researchers to contribute to the body of growing evidence and enable policymakers to make meaningful changes to support our global food system to make meaningful contributions to the Sustainable Development Goal 2: Zero Hunger. Based on the evidence generated from this review, the following recommendations are put forward to strengthen the evidence on the contribution of wild, NUS and forest foods to human diets and nutrition:

1. Move beyond ‘common foods only’ approach. Dietary intake assessments must document the vast number of food items known and consumed by local populations (including ethnobotanical and/or taxonomic assessments), before attempting to quantify their dietary contribution with contemporary intake assessments.
2. The dietary assessment method should be selected according to the research objective (e.g., identify qualitatively if wild, NUS, and forest foods are present in a diet vs. frequency and quantity of wild, NUS and forest consumption and resulting nutritional contribution to diets) and available resources (methods that are not resource and time consuming will probably be more widely used).
3. There is a need to develop and validate a suite of mixed-method diet recall approaches when assessing the importance of wild and NUS to diets, including using a hybrid or combination of 24HR and FFQ.
4. Dietary assessment methods need to be sufficiently detailed in scientific literature, including the foods used in those methods that use food lists in annexes or Supplementary material.
5. Advocacy is needed across the nutrition community on the importance of collecting, analysing and reporting dietary intake data in a manner that can reflect the contribution of wild, NUS and forest foods. This will help to empirically demonstrate their role which is needed to provide evidence-based policy recommendations that advocate for more biodiverse food systems for better nutrition.
6. There is a need for a global consultation to agree on standard terminologies to be used in dietary recall methods related to food sources and biodiversity related to NUS, forest, wild, local and traditional foods to promote better evidence generation.

Finally, the assessment of neglected forest and wild foods in diets will inform a careful consideration around the sustainable use of local foods in dietary guidelines. Various wild and underutilised species are current being overexploited and at risk of distinction or might introduce hazards related to zoonoses or food safety. Trade-offs on a sustainable use of food biodiversity, and the need to develop specific value chains, will need to be considered when developing guidance on the consumption of local food biodiversity in dietary guidelines.

## Conclusion

The studies identified in this scoping exercise were able to qualify that wild, forest and neglected foods do play an important part of diets in certain contexts. However, in most cases there was insufficient data or information presented from the diet recall methods used that would allow for a quantitative measure of their contribution to diet quality. Most studies utilised dietary recall methods that were unlikely to fully capture all wild, forest or neglected foods, even when they attempted to do so. One can then conclude that the true contribution of wild foods is probably underestimated. To overcome this, it is recommended that studies provide sufficient detail on their diet recall methods to allow for this assessment and that a specific diet recall methodology tool be developed and validated that is specifically designed to capture the presence and relative importance of these foods in diets. Doing so will decrease the resource constraints associated with such detailed dietary assessments, increasing the availability of more granular dietary intake data to enable a better understanding of how biodiverse foods can contribute to diet quality to help inform environmental, nutrition, health, and sustainability policy. If successful, and we do develop a more robust understating around the true contribution of wild foods we will then be faced with the challenge on how to utilise this information to sustainably integrate these foods more meaningfully into our food systems, whilst simultaneously minimising risk of overexploitation.

## Author contributions

CL and GH-C: designed the study. JR, JB, DC, and CL: conducted the research. JR: analysed data, performed statistical analysis, developed the first draft, and revised the manuscript. JB, GH-C, and CL: critically reviewed the manuscript. All authors contributed to the article and approved the submitted version.

## Funding

This work was supported by the Food and Agriculture Organization of the United Nations (FAO). The FAO contributed to the study design, interpretation of the findings and preparation of the manuscript, but were not involved in collection, analysis and decision to publish. Additional funding was provided by The Australian International Centre for Agriculture Research through supporting the time of JR to complete the manuscript.

## Acknowledgements

The authors thank Maria Antonia Tuazon (FAO) for her critical insights.

## Conflict of interest

The reviewer GK declared a past co-authorship and collaboration with the authors CL, GH-C, JR, and JB to the handling editor.

The remaining authors declare that the research was conducted in the absence of any commercial or financial relationships that could be construed as a potential conflict of interest.

## Publisher’s note

All claims expressed in this article are solely those of the authors and do not necessarily represent those of their affiliated organizations, or those of the publisher, the editors and the reviewers. Any product that may be evaluated in this article, or claim that may be made by its manufacturer, is not guaranteed or endorsed by the publisher.
