# Supplementary material for: Can common dietary assessment methods be better designed to capture the nutritional contribution of neglected, forest, and wild foods to diets?
Source: Front Nutr. 2023 Jul 6;10:1186707. doi: 10.3389/fnut.2023.1186707 (PMC10357295; doi:10.3389/fnut.2023.1186707)
Supplement: Supplementary file 1 [file Data_Sheet_1.docx]

**Supplementary Information**

**Supplemental Table 1. PubMed search and the number of titles retrieved^1^**

| **Scoping review** | **Syntax** | **Number of titles** |
| --- | --- | --- |
| Nutritional contributions of food from “forests” or “trees” | #1. (forest OR tree[mh])  #2. (diet[mh] OR nutrition[mh])  #3. (#1 AND #2)  #4. ((animals[mh]) NOT humans[mh])  #5. #3 NOT #4  #6. (editorial [pt] OR letter [pt] OR comment [pt])  #7. #5 NOT #6 | 690 |
| Nutritional contributions of “wild” foods or “NUS” | #1. (neglected OR wild)  #2. (diet[mh] OR nutrition[mh])  #3. (#1 AND #2)  #4. ((animals[mh]) NOT humans[mh])  #5. #3 NOT #4  #6. (editorial [pt] OR letter [pt] OR comment [pt])  #7. #5 NOT #6 | 1,660 |

^1^mh indicates that only articles were to be extracted with this topic as a major subheading. mh, MeSH heading; NUS, neglected and underutilized species.

**SI2 Data extraction template for full papers**

| Study (year) | Country | Population | # participants | Study design | Dietary assessment method | Foods assessed (forest, tree, wild, NUS) + definition (how are they defined in the paper) | Food classification | Outcome measure | Other pathways or food behaviours of wild/ forest food consumption |
| --- | --- | --- | --- | --- | --- | --- | --- | --- | --- |
|  |  |  |  |  |  |  |  |  |  |

**Supplemental Table S3. Screening of PubMed titles^1^**

| **Forest foods** | **Number of titles** |
| --- | --- |
| 1. PubMed search | 690 |
| 1. After title screening | 48 |
| 1. After abstract screening | 22 |
| 1. Full-text screening | 19 |
| 1. Final papers for full-text review | **9** |
| **Wild and NUS**^1^ **foods** | **Number of titles** |
| 1. PubMed search | 1660 |
| 1. After title screening | 110 |
| 1. Sought for retrieval | 93 |
| 1. Full-text screening | 93 |
| 1. Final papers for full-text review | **33** |
| **Forest, wild, and NUS**^1^ **Foods** | **Number of titles** |
| 1. PubMed search | 2,356 |
| 1. Articles sought for retrieval | 113 |
| 1. Full-text screening | 112 |
| 1. Final papers included in full-text review | 42 |
| 1. Final papers included in scoping study | **37** |

^1^NUS, neglected and underutilized species.

**SI4 Table Flood classification terms, outcome measure and summary of results.**

| **Study (year)** | **Food classification** | **Outcome measure** | **Summary of Results** |
| --- | --- | --- | --- |
| Ahenkan & Boon. (2011) | Non-Timber Forest Products (NTFP) (all biological materials other than timber which are extracted from forests, other wooded land, and trees outside forests and domesticated that include products used as food and food additives (edible nuts, mushrooms, grass-cutters, snails, fruits, herbs, spices and condiments, aromatic plants, game), fibres (used in construction, furniture, clothing, or utensils), resins, gums, and plant and animal products used for medicinal, cosmetic or cultural purpose for human use) | NTFP Consumption Frequency (Daily, Weekly, Occasionally, Rarely); nutrition (knowledge of), food security (undefined) | The respondents emphasized the importance of NTFPs, such as grass-cutters (Thryonomys swinderianus), antelopes, monkeys, snail, mushrooms, and fruits in their diets. The most widely consumed NTFPs were bushmeat, mushrooms, snails, leaves-food, honey, and fruits. The consumption of NTFPs among the poor households (5-6 day/week) was also greater than the middle-income groups (3 days/week). Relative importance of different NTFPs were graphically presented without quantifying the contribution; The percent of households that responded to to each frequency category per NTFP food item was present without quantification of contribution to diet quality. |
| Belahsen et al. (2017) | Food groups; wild edible plants (undefined), wild edibles (undefined) | Frequency of use of wild edible plants as food | No quantitative results presented on wild or forest food contribution to diet quality. Wild plants used as foods were reported has having a 30% frequency of use. 20 traditional recipes using these wild plants that are still frequently consumed, those consumed during periods of food shortage were stated as documented but not reported. |
| Belanger et al. (2010) | local Cultivated Green Leafy Vegetables (GLV) ; Wild foods: Uncultivated; wild GLV; Cultivation status (uncultivated, cultivated) | Consumption of cultivated and uncultivated/wild GLVs: mean servings/person/week, Carotenoid content of GLV, | Celosia argentea and Allmania nodiflora were the two wild species most frequently consumed. In total the weekly average number of GLV servings per person was 6.81±3.91, comprised of 4.59±3.00 servings/person/week of uncultivated species. One average portion was estimated to contains 20g of fresh leaves. There was a significant difference between the number of servings of cultivated and uncultivated GLV (p < 0.01). No significant difference was observed between the carotenoid contents of wild and commercially grown species. According to their reported frequency of consumption, the 10 species of GLV considered in this study contribute 40% of the daily recommended intake of β-carotene. The selected GLV species contributed to 1489 μg/day of β-carotene (980 μg from non-cultivated GLV) and 1788 μg/day of lutein (1186 μg from non cultivated GLV). |
| Benhura and Chitsaku (1992) | Food Groups; Home grown/cultivated; Wild(undefined); Semi-wild (normally grow in cultivated fields where they may be spared during hand cultivation. Some may grow on land on to which cattle manure has been applied) | Frequency in which various food items were consumed (% of meal including food item); number of meals consumed | A total of 146 food items were consumed. Wild and semi-wild vegetables and insects were consumed only in December/January when they were available. Eight out of seventeen leafy vegetables consumed were wild or semi-wild. No results presented on wild or forest food contribution to diet quality. |
| Blanchet et al. (2020) | The NOVA classification according to extent of processing; Traditional foods (wild fish, game, fowl, and plants) | Presence of traditional food in diet; % participants having consumed a traditional food mentioned in the 24h recall; % meeting DRIs; Healthy Eating Index (HEI-C): diet compared to 10 recommendations assessing 2 aspects of diet quality: adequacy and moderation. | 59 participants (22%) reported eating traditional foods. Traditional foods contributed to 13% of energy intake. Traditional food eaters had significantly higher intakes of protein; omega-3 fatty acids; dietary fibre; copper; magnesium; manganese; phosphorus; potassium; zinc; niacin; riboflavin; and vitamins B6, B12, D, and E than non-eaters. Traditional foods eaters also had significantly better diet quality based on the HEI-C. |
| Campbell (1986) | Wild fruits (undefined) ; Domesticated fruits (undefined) | Quantities of wild fruits in the diet | No household reported that wild fruits had been consumed in the 24-hour Period. Yet, over 95% of respondents in the stated that members of the family eat wild fruits. |
| do Nascimento et al. (2013) | Food plants: native (species of South-American origin); exotic (all of extra-continental origin); wild (those of spontaneous origin); cultivated | Number of people consuming certain species per season; total number of wild species consumed per season | Wild species consumption was higher in the dry season. The number of wild species consumed was significantly higher in Carão (5.75 ± 3.86) than in Cachoeira (3 ± 2.82 (p =.05). The low frequency of consumption of wild species suggests that these foods contribute little to contemporary dietary enrichment. |
| Dop et al. (2019) | wild edible plants (folk species - undefined) | % of women consuming wild edible plants over the year and median frequency of consumption (no of days/month) | No results presented on wild or forest food contribution to diet quality. 98% of women consumed a wild plant over the year with a median frequency of 2 days/month. |
| Dounias et al. (2007) | Food groups; wild(undefined) ; cultivated(undefined) | Origin of foods consumed ; Food intake over different periods of the year (g/day) per food category ;  Food intake (g/day) per food category. | The contribution of forest resources to the diet decreases with proximity to the city. The fat they use for cooking is mainly obtained from wild boar and is progressively replaced further downstream by manufactured palm oil. Among the Punan Benalui, meat contributes to 34% of the dishes; wild boar represents 86% of total game consumed. Fish or shellfish appear in only 15% of their dishes. |
| Fungo et al. (2016) | Forest foods(undefined) | Energy and nutrient intake , Dietary Diversity Score (DDS), Food Variety Score (FVS), Forest Food Consumption Score (FFCS) | Forty-seven unique forest foods were identified; of these, seventeen were consumed by 98% of respondents over the course of one week and by 17% of women. Although forest foods contributed approximately half of women’s total daily energy intake, considerably greater contributions were made to vitamin A (93 %), Na (100 %), Fe (85 %), Zn (88 %) and Ca (89 %) intakes. |
| Golden et al. (2011) | Domesticated meat (undefined) Wildlife meat (undefined) | Haemoglobin concentrations (of children <12 years) in response to wildlife consumption | Children who consumed a greater quantity of wildlife had higher haemoglobin concentrations [β (95% confidence interval, CI) = 0.20 (0.0078, 0.39), P = 0.041], when controlling for domesticated meat consumption. Removing access to wildlife would induce a 29% increase children suffering from anemia and a tripling of child anemia in the poor |
| Golden et al. (2019) | Food groups, wild meat (undefined) | Total mass of food consumption; % of energy requirements (EAR); % of nutrient intake (EAR); # of food items recorded in each category in the daily household diet records; biochemical parameters: micronutrient status (Hb, vitamin A, vita min B12, Fe, Zn, ferritin, transferrin receptor), calculated intake as % of EAR; HDDS; FCS; and MDD-W | Approximately 5.3% of all food consumed by weight was from animal-source foods, with wildlife contributing 40% of that value. Approximately 10.2% of all energy comes from animal-source foods. Of animal-source energy, 28.9 % comes from wild meat and fish. Wild meats and fish provide 16.9 % of protein, 5.8 % of Fe, 4.7 % of Zn, 5.2 % of PUFA, 16.2 % of Ca, 64.7 % of vitamin B12 and 71.7 % of consumed vitamin D. Total number of wild foods recorded are as follows: Bush meat: 22; Dried seafood: 5; Dried freshwater fish: 1; Freshwater fish: 5; Insects: 6; Seafood: 5; Wild birds: 24 |
| Kaufer et al. (2010) | Imported foods, locally-grown foods (undefined) | DDS; species diversity score; food variety score; contribution of imported and local foods to energy and nutrient intakes | No results presented on wild or forest food contribution to diet quality. Results of an intervention promoting local food consumption indicated a (110% increase in provitamin A carotenoid intake; increased frequency of consumption of local banana (53%), giant swamp taro (475%), and local vegetables (130%); and increased dietary diversity from local food. |
| Kent and Dunn (1996) | Wild meat (undefined) | Frequency of meat consumption (including wild meat) | The majority of families in 1989 consumed wild meat at least 2-3 times/week including scrub hare, wild cat, bat-eared fox, steebok, duiker and gemsbok. |
| Kolahdooz (2014) | Traditional foods (those from land, sea, sky) ; Non-nutrient-dense store-bought foods (NNDF) | Mean nutrient intake; dietary inadequacy (% below EAR); differences in nutrient density among men and women; top 10 foods contributing to energy and nutrient intakes | Traditional foods contributed considerably to protein and iron intake, and NNDF are the top contributors to energy, fat, carbohydrate, sugar, fiber, and sodium intake. Traditional foods from the land were the top contributors to protein (28%) and iron (33%) intake. Total Traditional foods contributed to 11% of the total energy consumed and 41% of total protein intake. |
| Kruger et al. (2008) | Green leafy plants; herbs; fish | Food Coping Strategy index, based on severity and frequency of food coping strategies; | No results presented on wild or forest food contribution to diet quality. Food gathering (as part of the food-seeking strategy) proved to play a major role in the relief of food stress in rural areas, where it is still possible to utilize the natural environment for food. |
| Liberda et al. (2018) | Market (purchased), Traditional (hunted, fished, foraged) foods. | Blood lead values; consumption frequency of traditional and market foods | Elevated blood lead levels were observed in association with increased hunting status and consumption of traditional foods. The first principal component extracted 9.8% of the total variance and was a clear summary of traditional food consumption: various species of wild-caught fish, waterfoul and wild berry jam all loaded strongly on principal component. |
| Manios et al. (2005) | Wild greens (undefined) | Mean daily energy and nutrient intake | During winter time consumption of wild greens increased in consumption. No results presented on wild or forest food contribution to diet quality. |
| Mansuri et al. (2016) | Traditional (undefined)  Balanced market foods (undefined) Healthier market foods (undefined) Western diet market foods (undefined) Wild (undefined) | Contribution of traditional foods (wild fish, in particular) to Vitamin D status. Serum 25(OH)D status; consumption frequency of 36 foods | Multivariate determinants of higher serum 25(OH)D included higher consumption of traditional and healthier market foods and higher wild fish consumption. Vitamin D blood concentrations were significantly positively correlated with diets based around traditional foods (fish, mosse meat, duck or goose, rabbit, Indian medicine or tea, home-made soup and wild berries). |
| M'Kaibi et al. (2015) | Food plants grown, Animals reared for food,  Food items obtained from natural habitats | NAR (RNI); MAR; | Only 3 (out of 26 foods sourced) were from the natural habitat. No results presented on wild or forest food contribution to diet quality. Household Agricultural biodiversity was positively and significantly related to all NARs (Spearman, p < 0.05) and MAR (Spearman, p < 0.001) indicating a significant positive relationship between agricultural biodiversity of the household with dietary adequacy of the child’s diet. |
| Ndaba and O'Keefe (1985) | Foodstuffs: meat; eggs; fish; fresh milk; sour milk; maize meal; bread; green vegetables; crushed maize with beans; rice ; Wild (undefined) | Frequencies of intake of various foodstuffs. | No results presented on wild or forest food contribution to diet quality. Wild spinach and cabbage was consumed daily during the summer rainfall months. |
| Ntwenya et al. (2017) | Wild foods (undefined) | Food Biodiversity Score (FBS): sum of different types of foods consumed by household | No results presented on wild or forest food contribution to diet quality. A total of 183 food items were reported by the households. The wild food consumption were reported by less than 10% of households. |
| Oduor et al. (2019) | Food groups | % of children consuming foods from different food groups; prevalence of Children meeting minimum diet diversity score; mean probability of adequacy (MPA); | No results presented on wild or forest food contribution to diet quality. A total of 80 species were maintained or harvested from the wild by the households. Mean household species richness was 9.9 ± 4.3. One in every four children did not meet the minimum dietary diversity score. The average mean probability of micronutrient adequacy was 68.11 ± 16.08 in plenty season compared to 56.37± 19.5% in the lean season. Iron, zinc and calcium were most limiting micronutrients in the diet, with less than 30% average probability of adequacy in both seasons. Household agrobiodiversity was positively associated with both dietary diversity score (r = 0.09, p = 0.029) and micronutrient adequacy (r = 0.15, p<0.000) in the pooled sample. One unit increase in species diversity was associated with 12.7% improvement in micronutrient adequacy. |
| Ogle et al. (2001) | Food groups | Micronutrient contribution from wild vegetables. Food Variety Score (FVS); DDS according to Hatloy et al. (2000): count of different food groups (12 in total; biochemical parameters: haemoglobin, serum ferritin, serum retinol, retinol binding protein, C-reactive protein. | A large variety of vegetables were consumed, approximately half of vegetable species were cultivated. Wild vegetables contributed significantly to the overall micronutrient intakes, women who consumed a greater variety and larger quantities of wild vegetables, also had significantly higher proportions of their iron and carotene intakes from wild vegetables. Contribution of wild foods to diet not quantified. |
| Penafiel et al. (2011) | Food sources: collected/hunted; cultivated; purchased but not processed; purchased or processed food; food aid ; Traditional foods: including both locally cultivated species and wild species (wild undefined); Local: collected, hunted or cultivated in the immediate geographic location | Nutrient Adequacy Ratios, Mean Adequacy Ratio (MAR), Dietary Species Richness (DSR) and Minimum Dietary Diversity for Women (MDDW), traditional food diversity score | Consumption of traditional foods contributed 38·6 % of total energy intake. Traditional Food diversity Score was associated with an increase in median MAR for macronutrients of 0·033 (P < 0·001) and an increase in median MAR for micronutrients of 0·052 (P < 0·001). Diets were based on 140 different food items, from which half were locally cultivated and wild species. About six traditional food species are consumed daily from local and wild sources. |
| Powell et al. (2013) | Food groups; Wild foods from farm; wild foods from forest (includes bush, river or other uncultivated land), Purchased foods, farm, gifts, foods from forest or uncultivated land. Wild foods: spontaneous growing/uncultivated. | 7-day Food Variety Score (FVS); 7-day dietary diversity score (DDS); 1-day FVS and DDS; contribution of foods from each source to diet (over 7 days) and nutrient intake. RDI% of energy and nutrients | Wild foods used by all informants (98.3% in the wet season and 93% in the dry season). 92 species of wild foods were reported . 26 foods were primarily (> 50% of times used) obtained from the forest, 45 were obtained from the forest a minimum of 10% of the time. The largest category of wild foods species (from any source) was vegetables. In the wet season 94% of mothers and 92% of children consumed one or more wild vegetable in the previous week (mean number of species was 4.1 ± 2.8 for mothers and 4.0 ± 2.8 for children). Wild foods contributed only 2% of total energy in the diet. However, they contributed large percentages of vitamin A (RAE) (31%), vitamin C (20%), and iron (19.19 %). Wild foods from forests contributed less than 1% of most nutrients in the diet (from 0.33% of energy to 1.3% of protein) |
| Powell et al. (2014) | Wild leafy vegetables (undefined) | Number of days (out of 7) household has consumed wild leafy vegetables | No results presented on wild or forest food contribution to diet quality. In Chachia and Sidi Sinoun 78% and 77% of households reported having used WLVs in the past 7 days. |
| Rao et al. (1993) | Food groups | % of RDA | No results presented on wild or forest food contribution to diet quality. No results presented on outcome measure. Preschool children suffered from low food and nutrient intake from all districts. Wild fruits were reported as consumed. |
| Robinson & Remins. (2016) | Wild meat, fish, payo  and other non-meat forest products | % of wild food relative to total food consumed | 35% of women reported eating wild game meat, 11% ate fish, while 100% consumed gozo (bitter manioc) and 95% consumed “payo” nuts (Irvingia wombolu). Approximately 71% of women consumed other nonmeat forest products, ranging from 1 to 5 different food items primarily koko (Gnetum africanum), but also mushrooms, yams, and wild fruits. No statistically significant differences were observed in the reported frequency of consumption of wild meat, fish, gozo, payo nuts, or other forest products between study villages. Foragers still use foraging to obtain the wide diversity of foods in their diet (about 70% of the food items identified during dietary recalls). |
| Roche et al. (2017) | Traditional foods (undefined) ; Wild leafy greens (undefined) | Estimated micronutrient contribution of two wild leafy greens to RDI | The wild leafy greens were estimated to contribute an additional 8% vitamin A, 7% iron, 12% vitamin C, and 27% folate to children's recommended dietary intakes. |
| Skreden et al. (2018) | Nordic' Foods (food groups and cooked foods); foods from the wild countryside | New Nordic Diet (NND) Score; | No results presented on wild or forest food contribution to diet quality. |
| Tata et al. (2019) | Food groups ; From the forest | MDD-W, anemia | The results of this study suggest that plant foods from the forest may make important contributions to iron intake and reduce the risk of anemia. Compared to women from grassland villages, women from the forest-based villages were more likely to have consumed vitamin A rich fruits and vegetables (98% vs. 92%; p = 0.04), nuts and seeds (88% vs. 46%; p<0.01), and meat and fish (84% vs. 68%; p<0.01). Nuts and seeds from the forest included bush mango (Irvingia garbonensis, irvingia wumbolo) and Njansang (Ricinodendron heudelotii). Compared to women from forest-based villages, women from the non-forest villages were more likely to have consumed ‘other’ vegetables e.g. tomato and okra (50% vs. 22%; p<0.01), and pulses (29% vs. 18%; p = 0.03). There was no difference in dietary diversity score between forest and non-forest women. |
| Taylor et al. (2013) | Game birds | Prevalence of game bird consumption; portion size and proportion of game bird meat of total meat intake in the UK | Fifty-eight participants (2.7 %) reported eating game birds. The mean intake was 19.5 (SD 18.1) g/d (median 15.6, range 1.3–92.9 g/d). In women of childbearing age (15–45 years), 11/383 (2.9 %) reported eating game birds, with a mean intake of 22.4 (SD 25.8) g/d (median 15.6, range 2.0–92.9 g/d). In children aged ≤6 years old, 3/342 (0.9 %) were reported as eating game birds, with a mean intake of 6.8 (SD 9.7) g/d (median 2.4, range 1.3–23.2 g/d). |
| Termote et al. (2012) | Food groups; Wild Edible Plants (WEPS - undefined) | Consumption frequency of food groups and WEPs; energy contribution of WEPs; dietary intake of WEP consumers and non-consumers and %women < RDA in both groups; | Results showed that in a high biodiverse region with precarious food security, WEP are insufficiently consumed to increase nutrition security or dietary adequacy. The highest contribution came from Dacryodes edulis in the village sample contributing 4.8% of total energy intake. |
| Termote et al. (2014) | Wild plant (undefined), Wild species (undefined) | Potential of 5 priority wild plant species to contribute to a cost reduction in relation to the nutritional gain (nutrient adequacy) of theoretically modeled diets using linear programming. | The modeled diets without wild species were deficient in iron for all age groups during the dry season, deficient in vitamin B6 and calcium for infants aged 6 to 8 months during the dry season, and deficient in iron and zinc for infants aged 6 to 8 months over the whole year. Adding wild foods, especially Berchemia discolor, to the modeled diets resulted in a lower-cost diet, while meeting recommended iron intakes for women and children between 12 and 23 months of age. |
| van Dijk et al (2003) | Non-Timber Forest Products (NTFP); edible mushrooms/fungal species | Average annual consumption quantity of mushroom; mushroom contribution to protein intake | All households collected edible fungi. Only in exceptional cases do mushrooms make a more substantial contribution. Average annual mushroom consumption by individuals is between 1.1 -1.4 kg (fresh weight). Most households (more than 90%) consume less than 3 kg fresh mushrooms per person per year. The relative contribution of mushrooms as a source of protein in the diet of both groups is small (this was not quantified). The major activities that yield protein are hunting and fishing (75 kg/yr for Bantu, 110 kg/year for Bagyeli). |
| Wesche and Chan (2010) | Traditional food (undefined) ; Fish, sea mammals, birds and land plants | Frequency of traditional food  consumption by species; estimated total intake of traditional food (g/person/day) by sex and age group | Caribou provides a large (22.2%) portion of the total energy intake from traditional foods.  All studied communities consume different types of fish and sea mammals, whereas birds and land plants show more selective importance. |

## **SI5 - Articles with (semi) Quantitative results on wild, nus and forest foods to diets**

| **Article** | **Definition of foods used** | **Quantitative contribution to diet reports** |
| --- | --- | --- |
| Belanger et al (2010) | wild species | 10 wild species contribute 40% of the daily recommended intake of β-carotene. |
| Blanche et al (2020) | traditional foods (included wild but did not specify if cultivated were included) | Traditional food eaters had significantly higher intakes of protein; omega-3 fatty acids; dietary fibre; copper; magnesium; manganese; phosphorus; potassium; zinc; niacin; riboflavin; and vitamins B6, B12, D, and E than non-eaters. Traditional food eaters also had significantly better diet quality based on the HEI-C. |
| Fungo et al 2016 | forest food | contributed to half of women’s total daily energy intake, vitamin A (93 %), Na (100 %), Fe (85 %), Zn (88 %), and Ca (89 %) intakes |
| Golden et al (2011) | wildlife | Children who consumed a greater quantity of wildlife had higher haemoglobin concentrations [β (95% confidence interval, CI) = 0.20 (0.0078, 0.39), P = 0.041], when controlling for domesticated meat consumption. Removing access to wildlife would induce a 29% increase children suffering from anaemia and a tripling of child anaemia in the poor |
| Golden et al (2019) | wildlife | that wildlife consumption contributed to 2.12% of food weight consumed and that 3% of all energy came from wild meat and fish. The authors also reported that wild meats and fish provide 16.9 % of protein, 5.8 % of Fe, 4.7 % of Zn, 5.2 % of PUFA, 16.2 % of Ca, 64.7 % of vitamin B12 and 71.7 % of consumed vitamin D. |
| Kaufer et al. (2010) | Imported foods, locally-grown foods (undefined) | No results presented on wild or forest food contribution to diet quality. Results of an intervention promoting local food consumption indicated a (110% increase in provitamin A carotenoid intake; increased frequency of consumption of local banana (53%), giant swamp taro (475%), and local vegetables (130%); and increased dietary diversity from local food. |
| Kolahdooz (2014) | Traditional foods (those from land, sea, sky) ; Non-nutrient-dense store-bought foods (NNDF) | Traditional foods contributed considerably to protein and iron intake, and NNDF are the top contributors to energy, fat, carbohydrate, sugar, fibre, and sodium intake. Traditional foods from the land were the top contributors to protein (28%) and iron (33%) intake. Total Traditional foods contributed to 11% of the total energy consumed and 41% of total protein intake. |
| Liberda et al. (2018) | Market (purchased), Traditional (hunted, fished, foraged) foods. | Elevated blood lead levels were observed in association with increased hunting status and consumption of traditional foods. The first principal component extracted 9.8% of the total variance and was a clear summary of traditional food consumption: various species of wild-caught fish, waterfoul and wild berry jam all loaded strongly on principal component. |
| Mansuri et al (2016) | traditional, healthy market and wild fish | Vitamin D blood concentrations were significantly positively correlated with diets based on traditional foods (need to get the specific increase from the paper) |
| Ogle et al (2001) | wild vegetables | women who consumed a greater variety and larger quantities of wild vegetables, also had significantly higher proportions of their iron and carotene intakes from wild vegetables |
| Penafiel et al (2011) | traditional (which included both cultivated and wild) | an increase in median mean nutrition adequacy ratio (MAR) for macronutrients of 0·033 (P < 0·001) ; increase in median MAR for micronutrients of 0·052 (P < 0·001) |
| Powell et al (2013) | wild forest foods | total dietary energy (2%), vitamin A (RAE) (31%), vitamin C (20%), and iron (19.19 %). 39.3% of protein, 27.6% of vitamin C, 26.7 % of iron, 25.6% of vitamin A (RAE), 23.2% of calcium intakes |
| Roche et al (2017) | wild leafy greens | contribute an additional 8% vitamin A, 7% iron, 12% vitamin C, and 27% folate to children's recommended dietary intakes |
| Termote et al (2012) | wild edible plants | one edible species providing a maximum of 5% of daily energy intake, wild edible plant consumption was insufficient to be a significant contribution to food or nutrition security |
| Termote et al (2014) | wild foods | adding wild foods, especially Berchemia discolor, to diets resulted in a lower-cost diet, while meeting recommended iron intakes for women and children between 12 and 23 months of age. |
| Wesche and Chan (2010) | traditional, wild animal | 22%) of energy intake consumed from traditional foods specifically came from a wild animal, |
